# Low Virulence and Lack of Airborne Transmission of the Dutch Highly Pathogenic Avian Influenza Virus H5N8 in Ferrets

**DOI:** 10.1371/journal.pone.0129827

**Published:** 2015-06-19

**Authors:** Mathilde Richard, Sander Herfst, Judith M. A. van den Brand, Pascal Lexmond, Theo M. Bestebroer, Guus F. Rimmelzwaan, Marion Koopmans, Thijs Kuiken, Ron A. M. Fouchier

**Affiliations:** Department of Viroscience, Postgraduate School Molecular Medicine, Erasmus MC, Rotterdam, The Netherlands; The University of Hong Kong, HONG KONG

## Abstract

Highly pathogenic avian influenza (HPAI) H5N8 viruses that emerged in poultry in East Asia spread to Europe and North America by late 2014. Here we show that the European HPAI H5N8 viruses differ from the Korean and Japanese HPAI H5N8 viruses by several amino acids and that a Dutch HPAI H5N8 virus had low virulence and was not transmitted via the airborne route in ferrets. The virus did not cross-react with sera raised against pre-pandemic H5 vaccine strains. This data is useful for public health risk assessments.

## Introduction

Highly pathogenic avian influenza (HPAI) H5N1 influenza viruses of the A/Goose/Guangdong/1/1996 (GsGd) lineage were first detected in poultry in Hong Kong in 1997 and became enzootic in poultry in many countries of Africa, Europe and Asia since 2003. The continued circulation of HPAI H5N1 viruses in poultry led to the genetic diversification of the hemagglutinin (HA) gene into multiple genetic clades, but until 2009, there was no evidence of reassortment events between HPAI H5N1 viruses and other influenza viruses [1]. However, from 2009 onwards, HPAI viruses of subtypes H5N2, H5N5, H5N6 and H5N8, containing the H5 gene of the GsGd lineage, the neuraminidase (NA) and various other genes of LPAI virus origin, were detected in poultry [2–5].

HPAI H5N8 viruses were first detected in domestic birds in China in 2010 and emerged early 2014 in poultry in the Republic of Korea and Japan [4, 6]. In late 2014, the HPAI H5N8 viruses spread to Europe and North America, where they were detected in domestic birds, wild birds or both [7, 8]. The gene constellation of HPAI H5N8 viruses isolated in the Republic of Korea, Japan, Europe and North America was similar. They possess HA, NA, basic polymerase 2 (PB2) and nucleoprotein (NP) gene segments derived from HPAI H5N8 viruses isolated in China in 2010 [4] and the remaining genes derived from low pathogenic avian influenza (LPAI) viruses isolated in China in 2011 [9]. The HA and PB2 genes cluster with genes of viruses of the clade 2.3.4.4 of the HPAI H5N1 GsGd lineage [10].

No human cases of infection with HPAI H5N8 virus have been reported to date. However, since HPAI H5N1 viruses with HA of the GsGd lineage have previously infected humans [11], it is important to assess the potential public health risk upon human exposure to HPAI H5N8 virus and to evaluate intervention options. It was previously shown by Kim *et al*. that a Korean HPAI H5N8 strain had low virulence in ferrets and did not transmit via the airborne route or through direct contact. However, since the viruses that were isolated in Europe possess several amino acid differences compared to those isolated in the Republic of Korea and in Japan, we sought to study the virulence, transmissibility and antigenicity of the Dutch HPAI H5N8 virus A/Chicken/Netherlands/EMC-3/2014 in ferrets.

## Material and Methods

### Cells

Madin-Darby canine kidney (MDCK) cells (ATCC) were cultured in Eagle’s minimal essential medium (EMEM, Lonza Benelux BV, Breda, the Netherlands) supplemented with 10% fetal bovine serum (FBS) (Greiner), 100U/ml penicillin (P, Lonza), 100U/ml streptomycin (S, Lonza), 2mM L-glutamine (L-glu, Lonza), 1.5mg/ml sodium bicarbonate (NaHCO_3_, Lonza), 10mM Hepes (Lonza) and 1X non-essential amino acids (NEAA, Lonza).

### Viruses

A/Chicken/Netherlands/EMC-3/2014 virus was isolated from a lung homogenate of a naturally infected chicken during an outbreak in Ter Aar, The Netherlands. The virus was then propagated in MDCK cells. Recombinant A/Indonesia/5/2005 virus was generated as previously described [12].

### Virus titration in MDCK cells

Virus titrations were performed as described previously [13]. Briefly, MDCK cells were inoculated with tenfold serial dilutions of virus stocks, nose swabs, throat swabs or homogenized tissue samples. Cells were washed with PBS one hour after inoculation and cultured in infection media, consisting of EMEM supplemented with 100U/ml P, 100U/ml S, 2mM L-glu, 1.5mg/ml NaHC0_3_, 10mM Hepes, 1X NEAA and 20μg/ml trypsin (Lonza). Three days after inoculation, supernatants of cell cultures were tested for agglutinating activity using turkey erythrocytes as an indicator of virus replication. Infectious virus titers were calculated from four replicates each of the homogenized tissue samples, nose swabs and throat swabs and from ten replicates of the virus stocks by the method of Reed and Muench [14].

### Ethical statements

Animals were housed and experiments were conducted in strict compliance with European guidelines (EU directive on animal testing 86/609/EEC) and Dutch legislation (Experiments on Animals Act, 1997) (S1 ARRIVE Checklist). All animal experiments were approved by the independent animal experimentation ethical review committee ‘stichting DEC consult’ (Erasmus MC permit number EUR3385). The DEC considers the application and pays careful attention to the effects of the intervention on the animal, its discomfort and weighs this against the social and scientific benefit to humans or animals. The researcher is required to keep the effects of the intervention to a minimum, based on the three Rs (Refinement, Replacement, Reduction). Animal welfare was monitored on a daily basis. Virus inoculation of ferrets was performed under anesthesia with a mixture of ketamine/medetomidine (10 and 0.05 mg/kg resp.) antagonized by atipamezole (0.25 mg/kg). All animal handling (swabbing and weighing) was performed under light anesthesia using ketamine to minimize animal suffering. Influenza virus and Aleutian Disease Virus seronegative 6-month-old female ferrets (*Mustella putorius furo*), weighing 700–1000 g., were obtained from a commercial breeder. All experiments with ferrets were performed under animal biosafety level 3+ conditions in class 3 isolator cages.

### Pathogenesis in ferrets

Six ferrets were inoculated intranasally with 10^6^ median tissue culture infectious doses (TCID_50_) of HPAI H5N8 A/Chicken/Netherlands/EMC-3/2014 or HPAI H5N1 A/Indonesia/5/2005. Clinical scores in all groups were assessed every day. Activity status was scored as follows: 0, alert and playful; 1, alert and playful only when stimulated; 2, alert but not playful when stimulated; 3, neither alert nor playful when stimulated. Dyspnea was characterized by open-mouth breathing with exaggerated abdominal movement [15]. For diarrhea, sneezing, nasal discharge, inappetence and dyspnea we scored: 0, not present; 1, present. Body weight was monitored daily. Throat and nose swabs were collected every day and were stored at -80°C in transport medium (Hank’s balanced salt solution containing 0.5% of lactalbumin (Sigma), 10% of glycerol (Sigma), 200U/ml P, 200 mg/ml S, 100U/ml polymixin B sulphate (Sigma) and 250 mg/ml gentamicin (Gibco)) for end-point titration in MDCK cells. At 3 and 6 days post infection (dpi), three animals from each group were euthanized by exsanguination under anesthesia and were necropsied according to a standard protocol [15]. Tissues harvested for virological examination were homogenized in transport medium using the FastPrep system (MP Biomedicals) with 2 one-quarter-inch ceramic sphere balls, centrifuged 1500x*g* for 10 min, aliquoted and stored at -80°C for end-point titration in MDCK cells. Tissues harvested for histological examination were fixed in 10% neutral-buffered formalin, embedded in paraffin, sectioned at 4 μm and stained with hematoxylin and eosin (HE) for examination by light microscopy.

### Immunohistochemistry (IHC)

For detection of influenza A virus antigen, sequential slides of all tissues were stained with a primary antibody against the influenza A NP as described previously [15]. In each staining procedure, an isotype control was included as a negative control and a lung section from a cat infected experimentally with H5N1 was used as positive control [16].

### Transmission experiment

Airborne transmission experiments were performed as described previously [12, 13]. In short, four ferrets were inoculated intranasally under anesthesia with 10^6^ TCID_50_ of virus by applying 250 μl of virus suspension to each nostril. Each donor ferret was then placed in a transmission cage. One day after inoculation, one naïve recipient ferret was placed opposite of each donor ferret. Each transmission pair was housed in a separate transmission cage designed to prevent direct contact but to allow airflow from the donor to the recipient ferret. Nose and throat swabs were collected at 1, 3, 5 and 7 dpi from donor ferrets and at 1, 3, 5, 7 and 9 days post exposure (dpe) from the recipient ferrets and subjected to virus titration. Donor ferrets were euthanized at 7 dpi and recipient ferrets at 14 dpe to allow assessment of seroconversion.

### Hemagglutination inhibition (HI) assay

Ferret antisera were prepared as previously described [17]. Ferret antisera were then pre-treated overnight with receptor destroying enzyme (*Vibrio cholerae neuraminidase*) at 37°C and incubated at 56°C for 1h the next day. Twofold serial dilutions of the antisera, starting at a 1:20 dilution, were mixed with 25μl of a virus stock containing 4 hemagglutinating units and were incubated at 37°C for 30 minutes. Subsequently, 25μl of 1% turkey erythrocytes was added and the mixture was incubated at 4°C for 1h. HI was read and was expressed as the reciprocal value of the highest dilution of the serum that completely inhibited agglutination of virus and erythrocytes.

## Results and Discussion

To assess the zoonotic potential of HPAI H5N8 viruses, we searched for known mammalian adaptation substitutions in the sequences of HPAI H5N8 viruses available in GISAID (13 strains from Europe and 41 strains from Korea and Japan, S1 Table) using the H5N1 genetic changes inventory from the Centers for Disease control and Prevention [18]. Based on our knowledge of HPAI H5N1 viruses, we found 8 substitutions in the HPAI H5N8 sequences, which may influence receptor binding preference, virulence and antiviral susceptibility (Table 1). Moreover, we performed a comparison of the sequences of HPAI H5N8 viruses from Europe and Korea/Japan, which revealed several amino acid differences between the European strains and the Korean and Japanese strains (Table 2). Two of these substitutions, R699K in PB2, which is located next to the well-known host adaptation substitution D701N [19], and A185E in HA, which has previously been described to have an antigenic effect within the H5 clade 2.1 [17], warranted the evaluation of the virulence, transmissibility and antigenicity of the European HPAI H5N8 virus.

**Table 1:** Amino acid substitutions in HPAI H5N8 viruses from Europe and Asia that may affect receptor binding preference, virulence or susceptibility to antivirals.

Gene segment	Substitution	Phenotype	Reference
PB1-F2	N66S	Increased virulence, replication efficiency and antiviral response in mice	[24]
HA	S133A	Increased binding to α2.6 linked sialic acid receptors	[25]
	T156A	Deletion of a putative glycosylation site, increased virus binding to α2.6 linked sialic acid receptors and increased transmission in guinea pigs and in ferrets	[12, 26, 27]
M1	D30N	Increased virulence in mice	[28]
	T215A	Increased virulence in mice	[28]
M2	S31N	Reduced susceptibility to amantadine and rimantadine	[29]
NS1	P42S	Increased virulence in mice	[30]
	I101M	Increased virulence in mice	[31]

**Table 2:** Amino acid differences between Korean and Japanese HPAI H5N8 or European HPAI H5N8 strains.

Gene segment	Amino acid position	Amino acid in Korean and Japanese H5N8 strains	Amino acid in European H5N8 strains
PB2	338	V	I
	699	K	R
PB1	57	T	K
PA	162	T	I
	343	A	V
	385	R	K
HA	181	S	P
	185	A	E
	374	I	V
NP	51	D	N
NA	190	A	T
NS	65	V	L

In our study, we used, as a prototype for the European HPAI H5N8 viruses, a Dutch HPAI H5N8 A/Chicken/Netherlands/EMC-3/2014 virus, which was isolated from a lung homogenate of a naturally infected chicken during an outbreak in Ter Aar (The Netherlands) [20]. On the 19^th^ of November, it was reported to the Dutch Ministry of Economic Affairs that chickens in a farm located in Ter Aar were showing clinical signs of avian influenza infection. After laboratory confirmation of an outbreak of the avian influenza H5 subtype, the remaining chickens were culled the day after. In total, this outbreak led to the death of 43000 chickens.

**Fig 1:**
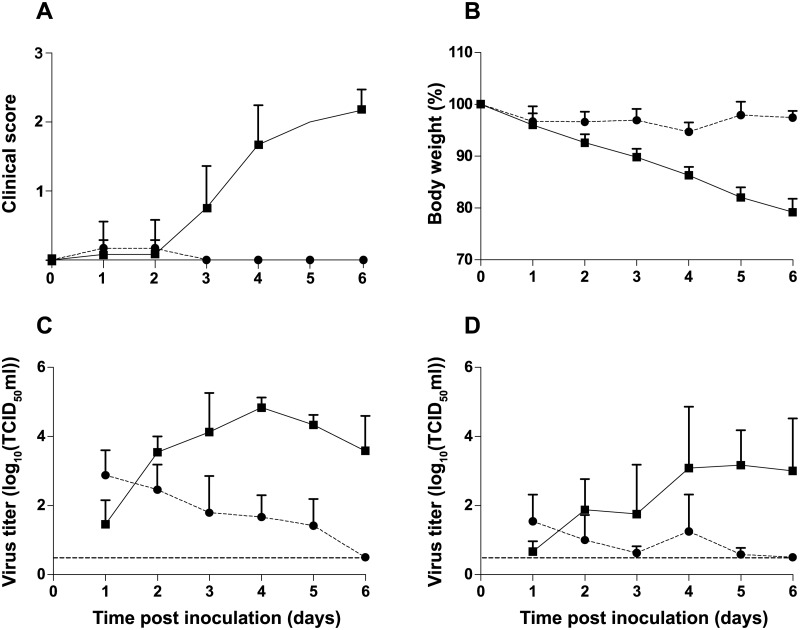
Average clinical scores, body weight and virus titers in ferrets upon intranasal inoculation with HPAI H5N8 virus A/Chicken/NL/EMC-3/2014 (circles) or HPAI H5N1 virus A/Indonesia/5/2005 (squares). **A.** Activity status was scored daily as follows: 0, alert and playful; 1, alert and playful only when stimulated; 2, alert but not playful when stimulated; 3, neither alert nor playful when stimulated. **B.** Body weight. **C.** Virus titers in the throat swabs. **D.** Virus titers in the nasal swabs. All titers were determined by end-point titration in MDCK cells. The lower limit of detection is indicated by the dashed lines. All error bars represent standard deviation.

**Fig 2:**
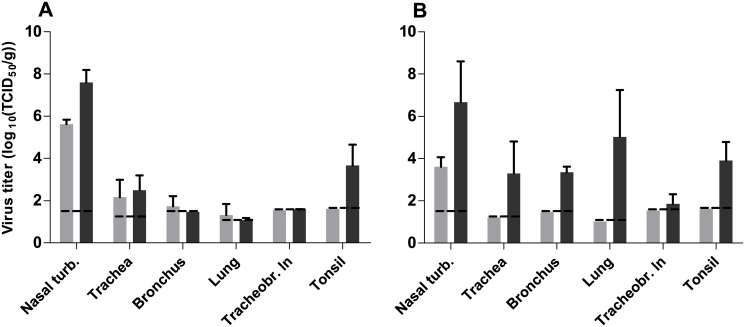
Virus titers in respiratory tract tissues of ferrets upon intranasal inoculation with HPAI H5N8 A/Chicken/NL/EMC-3/2014 (light grey) or HPAI H5N1 A/Indonesia/5/05 (dark grey). **A.** Average virus titers at 3 dpi. **B.** Average virus titers at 6 dpi. All titers were determined by end-point titration in MDCK cells. The lower limit of detection is indicated by the dashed lines. All error bars represent standard deviation. Nasal turb.: Nasal turbinates. Tracheobr. ln: Tracheobronchial lymph node.

**Fig 3:**
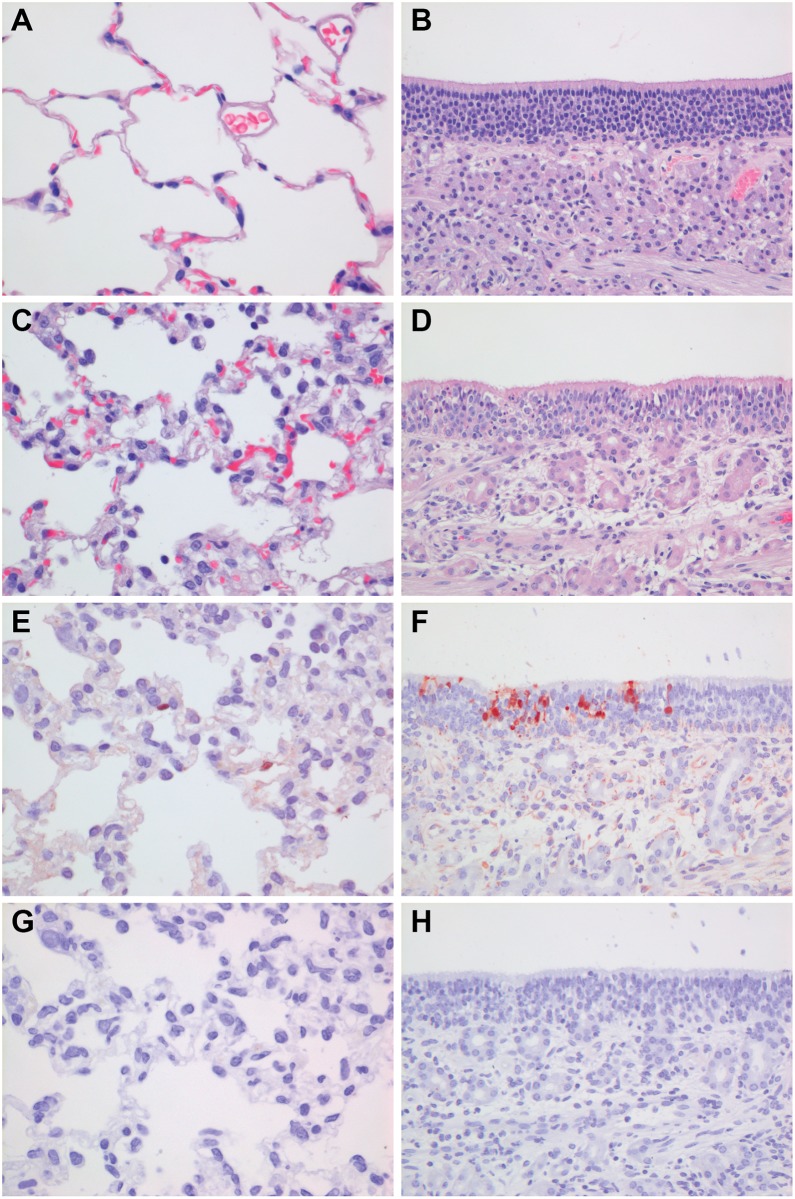
Histopathology and virus antigen expression in lung and nose tissues of ferrets upon intranasal inoculation with HPAI H5N8 A/Chicken/NL/EMC-3/2014 at 3 dpi. **A and B.** HE staining of a naïve ferret lung and nasal olfactory epithelium respectively. **C and D.** HE staining of lung and nasal olfactory epithelium from H5N8-inoculated ferrets respectively, showing mild necrosis and inflammation of the alveolar epithelium in the lung and olfactory epithelium in the nose associated with antigen expression. **E and F.** Influenza A virus nucleoprotein antigen as determined by IHC in alveolar epithelial cells in the lung and in olfactory epithelium in the nose of H5N8-inoculated ferrets respectively. **G and H**. Isotype controls corresponding to E and F.

Firstly, we compared the virulence of the A/Chicken/Netherlands/EMC-3/2014 virus, with that of HPAI H5N1 A/Indonesia/5/05 virus in ferrets. Ferrets have been used in influenza research since 1933 because they show similar susceptibility to infection with human and avian influenza viruses and develop respiratory disease similar to that observed in humans [13]. Upon intranasal inoculation of six ferrets with 10^6^ TCID_50_ of HPAI H5N8 virus, ferrets did not develop severe disease or display pronounced clinical signs. Ferrets had mild serous nasal discharge from 2 to 6 dpi and one animal was slightly less active at 1 and 2 dpi (Fig 1A). In contrast, ferrets inoculated with the HPAI H5N1 virus became less active from 3 dpi onwards and their condition deteriorated until 6 dpi, when animals were euthanized for ethical reasons. Inappetence and dyspnea were observed in all HPAI H5N1 virus-inoculated ferrets at 6 dpi. The mean maximum weight loss of the HPAI H5N8 virus-inoculated animals was 5%, substantially less than that of animals inoculated with HPAI H5N1 virus (>20%) (Fig 1B) or human influenza viruses (10% and 12% for seasonal and 2009 pandemic H1N1 viruses, respectively) [13]. Virus shedding from the throat and nose of HPAI H5N1 virus-inoculated ferrets peaked at 4 dpi, with titers up to 10^4.5^ TCID_50_, whereas virus shedding from HPAI H5N8-inoculated ferrets declined already after 1 dpi, when it only reached a mean maximum titer of 10^2.9^ TCID_50_ (Fig 1C and 1D). HPAI H5N8 virus was detected exclusively in the respiratory tract of ferrets, with lower virus titers compared to HPAI H5N1 virus (Fig 2). At 3 dpi, the virus titers in the nasal turbinates and tonsils were higher in HPAI H5N1 virus-inoculated animals. At 6 dpi, the differences in virus titers in the respiratory tract between HPAI H5N8 virus and HPAI H5N1 virus-inoculated animals were more pronounced, with higher titers for HPAI H5N1 virus in all six tissues collected from the respiratory tract. Virus was not detected in any of the extra-respiratory tract organs that were collected from HPAI H5N8 virus-inoculated animals. In contrast, HPAI H5N1 virus was detected in extra-respiratory organs, such as olfactory bulb, brain, jejunum, spleen and liver (data not shown). Low virus shedding from the respiratory tract was also reported for HPAI H5N8 virus by Kim *et al*. using A/mallard duck/Korea/W452/2014, but low amounts of virus were also detected in brain, spleen, liver and colon at 5 dpi in that study [9]. Gross pathology of the lungs of HPAI H5N8 virus-inoculated ferrets revealed multiple dark red areas in the lungs, consistent with consolidation ranging from 5 to 20% of the lung surface area at 3 dpi and from 5 to 10% at 6 dpi. At 3 dpi, influenza virus antigen expression was seen in two ferrets in a few alveolar epithelial cells that morphologically resembled type II pneumocytes and occasionally type I pneumocytes, with associated lesions characterized by focal to multifocal mild to moderate intra-alveolar edema with fibrin, few to moderate numbers of neutrophils and mild increase in the number of alveolar macrophages. There was mild necrosis of alveolar epithelial cells and mild to moderate hypertrophy of alveolar and bronchiolar epithelium (Fig 3). In another ferret, virus antigen was detected in many olfactory epithelial cells, associated with multifocal mild to moderate necrosis of olfactory epithelium with mild exocytosis of neutrophils (Fig 3). Influenza virus antigen expression was not seen in other parts of the respiratory tract or in extra-respiratory tissues at 3 dpi. At 6 dpi, two ferrets had mild mononuclear interstitial pneumonia with type II pneumocyte hyperplasia (regeneration) in the lungs with no virus antigen expression. There was also no virus antigen expression in any other collected tissues at 6 dpi. This contrasts with HPAI H5N1 virus-inoculated ferrets, where influenza virus antigen expression was observed in moderate numbers of type I and II pneumocytes, bronchiolar epithelial cells and in few alveolar macrophages at 6 dpi and in the nose in many olfactory and respiratory epithelial cells at 3 dpi and less at 6 dpi (data not shown).

**Fig 4:**
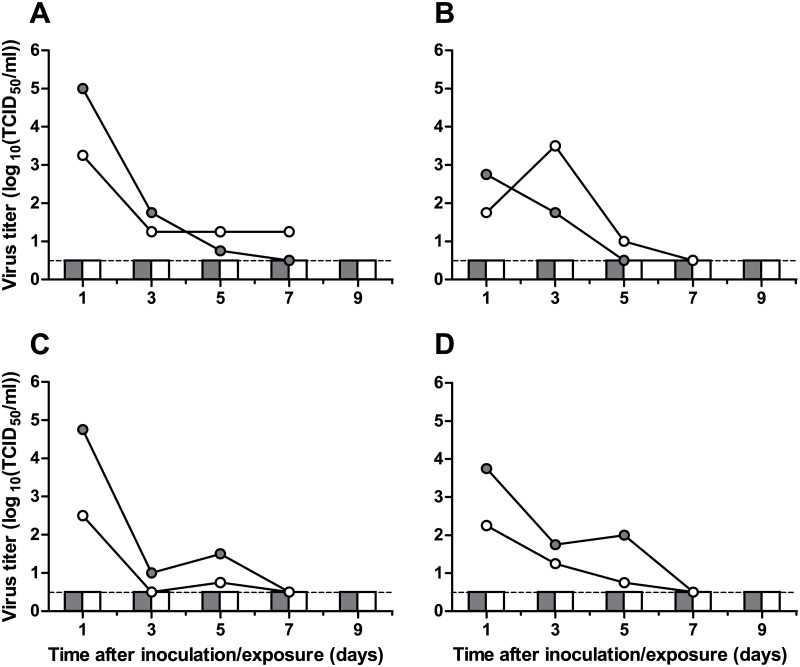
Lack of airborne transmission of HPAI H5N8 virus A/Chicken/Netherlands/EMC-3/2014 between ferrets. Data for individual transmission experiments is shown in each panel, with virus shedding from donor and recipient ferrets shown as lines and bars, respectively. Grey circles and bars represent shedding from the throat; white circles and bars represent shedding from the nose. The lower limit of detection is indicated by the dashed lines.

Because transmission via respiratory droplets and aerosols (hereafter referred to as “airborne transmission”) is the main route for influenza virus transmission between humans, it is of importance to investigate the airborne transmissibility of emerging influenza A viruses. In the inoculated donor ferrets, virus shedding was low and short-term (Fig 4). After peak virus shedding at 1 dpi for most animals, virus titers in both throat and nose swabs decreased rapidly. The HPAI H5N8 virus was not transmitted via the airborne route as demonstrated by the absence of replicating virus (Fig 4) and seroconversion (HI titers<10) in any of the four recipient ferrets. This is in accordance with other studies on transmissibility of HPAI H5N8 virus, which reported the lack of contact and airborne transmission of A/Mallard/Korea/W452/2014 between ferrets [9] and of contact transmission of A/Duck/Shandong/Q1/2013 and A/Duck/Jiangsu/k1203/2010 between guinea pigs [21].

Vaccination and antiviral therapy are the main options to prevent and treat human influenza virus infections. We evaluated the recognition of A/Chicken/Netherlands/EMC-3/2014 by ferret antisera raised against various pre-pandemic H5 vaccine strains. None of these older strains elicited HI antibodies that cross-reacted with the H5N8 HA, suggesting that these selected pre-pandemic H5 vaccine strains did not match antigenically (Table 3). Although several more recent H5 candidate vaccine strains are available, also these were reported to have a poor antigenic match [22]. A/mallard duck/Korea/W452/2014 was previously reported to be sensitive to oseltamivir, zanamivir and peramivir [9] and the amino acid substitution in NA that differs between the Korean/Japanese and European HPAI H5N8 viruses is not located in the NA active site, suggesting that above drugs can be used prophylactically or therapeutically if the need arises.

**Table 3:** HI assay with ferret antisera raised against a panel of candidate H5 vaccine viruses for pandemic preparedness selected by the WHO network.

	Ferret antisera raised against						
Virus	LPAI	Clade 0	Clade 1	Clade 2.1	Clade 2.2	Clade 2.3	H5N8
A/Mallard/Netherlands/3/1999 (H5N2, LPAI)^a^	160	120	<10	<10	10	<10	<10
A/Hong Kong/156/1997 (H5N1, Clade 0)	160	560	120	40	320	80	80
A/Vietnam/1194/2004 (H5N1, Clade 1)	<10	<10	80	<10	<10	<10	<10
A/Indonesia/5/2005 (H5N1, Clade 2.1)	<10	<10	<10	160	<10	<10	<10
A/Turkey/Turkey/1/2005 (H5N1, Clade 2.2)	40	20	10	<10	1280	40	<10
A/Anhui/1/2005 (H5N1, Clade 2.3)	<10	<10	20	40	40	160	<10
A/Chicken/Netherlands/EMC-3/2014 (H5N8)	<10	20	<10	<10	<10	<10	240

Underlined numbers show homologous HI titers.

^a^ LPAI: Low pathogenic avian influenza

Collectively, our results demonstrate that the Dutch HPAI H5N8 A/Chicken/Netherlands/EMC-3/2014 virus replicates poorly in ferrets, has low virulence and lacks the ability to transmit via the airborne route in the ferret model. This suggests that none of the amino acid substitutions found in the European HPAI H5N8 viruses, as compared to the Korean and Japanese HPAI H5N8 viruses, affects these properties. The marked difference in virulence between the HPAI H5N8 and HPAI H5N1 viruses might be due to their different gene constellations. All the internal genes of HPAI H5N1 viruses are derived from H9N2 influenza viruses, which also donated internal genes to other zoonotic influenza viruses, such as H7N9 and H10N8 viruses [23]. Except for the HA and PB2 gene segments, which are derived from the GsGd lineage, the gene segments of HPAI H5N8 viruses were acquired from LPAI viruses that circulate in wild birds, which may explain why this H5N8 lineage has evolved into a virus that is better adapted to wild birds, supporting its rapid geographical spread from Asia, to Europe and North America.

Careful extrapolation from animal models to humans suggests that the public health threat of the HPAI H5N8 strains is low. However, given the rapid geographical spread of HPAI H5N8 viruses, their ability to infect various species of birds and mammals and the propensity of influenza A viruses to mutate and reassort, the HPAI H5N8 viruses epizootics should be monitored closely.

## Supporting Information

S1 ARRIVE Checklist NC3Rs ARRIVE Guidelines Checklist. (PDF)

S1 Table List of the H5N8 viruses used for the genetic analysis. (DOCX)
