# Supplementary material for: Low Virulence and Lack of Airborne Transmission of the Dutch Highly Pathogenic Avian Influenza Virus H5N8 in Ferrets
Source: PLoS One. 2015 Jun 19;10(6):e0129827. doi: 10.1371/journal.pone.0129827 (PMC4474857; doi:10.1371/journal.pone.0129827)
Supplement: S1 Table — (DOCX) [file pone.0129827.s002.docx]

| H5N8 viruses from Europe | H5N8 viruses from Korea or Japan |
| --- | --- |
| A/domestic duck/Germany-NI/R3468/2014 | A/Korean_native_chicken/Korea/H257/2014 |
| A/turkey/Germany-NI/R3372/2014 | A/broiler_duck/Korea/H133/2014 |
| A/turkey/Italy/14VIR7898-10/2014 | A/broiler_duck/Korea/H47/2014 |
| A/chicken/Netherlands/emc-3/2014 | A/broiler_duck/Korea/H31/2014 |
| A/turkey/Germany/R2474-L00899/2014 | A/broiler_duck/Korea/H29/2014 |
| A/eurasian wigeon/Netherlands/emc-2/2014 | A/coot/Korea/H81/2014 |
| A/eurasian wigeon/Netherlands/emc-1/2014 | A/baikal_teal/Korea/H80/2014 |
| A/duck/England/36226/14 | A/baikal_teal/Korea/Donglim3/2014 |
| A/duck/England/36038/14 | A/Baikal_teal/Korea/H62/2014 |
| A/chicken/Netherlands/14015531/2014 | A/spot-billed_duck/Korea/H455-42/2014 |
| A/chicken/Netherlands/14015526/2014 | A/waterfowl/Korea/S005/2014 |
| A/duck/England/36254/14 | A/tundra_swan/Korea/H411/2014 |
| A/turkey/Germany-MV/R2472/2014 | A/broiler_duck/Korea/Buan2/2014 |
|  | A/breeder_chicken/Korea/H503/2014 |
|  | A/breeder_duck/Korea/H200/2014 |
|  | A/common_teal/Korea/H455-30/2014 |
|  | A/bean_goose/Korea/H40/2014 |
|  | A/breeder_duck/Korea/H249/2014 |
|  | A/mallard/Korea/H207/2014 |
|  | A/broiler_duck/Korea/H145/2014 |
|  | A/breeder_duck/Korea/H128/2014 |
|  | A/Baikal_teal/Korea/H66/2014 |
|  | A/bean_goose/Korea/H53/2014 |
|  | A/baikal_teal/Korea/H41/2014 |
|  | A/breeder_duck/Korea/H158/2014 |
|  | A/breeder_chicken/Korea/H122/2014 |
|  | A/baikal_teal/Korea/H84/2014 |
|  | A/baikal_teal/Korea/H68/2014 |
|  | A/broiler_duck/Korea/H65/2014 |
|  | A/bean_goose/Korea/H328/2014 |
|  | A/breeder_chicken/Korea/H250/2014 |
|  | A/white-fronted_goose/Korea/H231/2014 |
|  | A/baikal_teal/Korea/H96/2014 |
|  | A/broiler_duck/Korea/H49/2014 |
|  | A/broiler_duck/Korea/H48/2014 |
|  | A/mallard/Korea/H297/2014 |
|  | A/chicken/Kumamoto/1-7/2014 |
|  | A/mallard/Korea/W452/2014 |
|  | A/crane/Kagoshima/KU13/2014 |
|  | A/crane/Kagoshima/KU1/2014 |
|  | A/baikal_teal/Korea/H52/2014 |
|  |  |
|  |  |
|  |  |
|  |  |
